# *Npas4a* expression in the teleost forebrain is associated with stress coping style differences in fear learning

**DOI:** 10.1038/s41598-021-91495-7

**Published:** 2021-06-08

**Authors:** Matthew R. Baker, Ryan Y. Wong

**Affiliations:** 1 Department of Biology, University of Nebraska at Omaha, Omaha, USA; 2 Department of Psychology, University of Nebraska at Omaha, 6001 Dodge St, Omaha, NE 68182 USA

**Keywords:** Behavioural ecology, Neuroscience, Learning and memory, Stress and resilience

## Abstract

Learning to anticipate potentially dangerous contexts is an adaptive behavioral response to coping with stressors. An animal’s stress coping style (e.g. proactive–reactive axis) is known to influence how it encodes salient events. However, the neural and molecular mechanisms underlying these stress coping style differences in learning are unknown. Further, while a number of neuroplasticity-related genes have been associated with alternative stress coping styles, it is unclear if these genes may bias the development of conditioned behavioral responses to stressful stimuli, and if so, which brain regions are involved. Here, we trained adult zebrafish to associate a naturally aversive olfactory cue with a given context. Next, we investigated if expression of two neural plasticity and neurotransmission-related genes (*npas4a* and *gabbr1a*) were associated with the contextual fear conditioning differences between proactive and reactive stress coping styles. Reactive zebrafish developed a stronger conditioned fear response and showed significantly higher *npas4a* expression in the medial and lateral zones of the dorsal telencephalon (Dm, Dl), and the supracommissural nucleus of the ventral telencephalon (Vs). Our findings suggest that the expression of activity-dependent genes like *npas4a* may be differentially expressed across several interconnected forebrain regions in response to fearful stimuli and promote biases in fear learning among different stress coping styles.

## Introduction

Animals frequently must overcome stressors and the ability to encode and recall these salient experiences is essential to an individual’s survival. Within individuals, behavioral and physiological responses to stressors often co-vary, belonging to correlated suites of traits that are consistent across contexts and time^1–4^ (i.e. animal personality, stress coping styles; bold-shy axis, proactive–reactive axis). In addition to boldness, aggression, and stress physiology, studies demonstrate that proactive and reactive individuals also differ in learning and memory processes^5–9^. The more risk-prone proactive individuals tend to show faster acquisition of memories that require higher levels of activity, or paradigms with positive and rewarding valence^10–16^. In contrast, the risk-averse reactive individuals tend to show faster acquisition of aversive paradigms that require avoidance or reduced levels of activity^17–19^. Despite these findings, the neuromolecular mechanisms and regional brain activity underlying these stress coping style differences in learning are not well understood.

Recent work has suggested that neural plasticity and neurogenesis may be key mechanisms underlying divergent proactive–reactive responses to stress, but whether these processes are associated with differences in learning and memory is not understood^20,21^. While previous studies have characterized the whole-brain transcriptome of proactive and reactive individuals at baseline, the contribution of specific neural plasticity- and synaptic transmission-related candidate genes and their spatial expression patterns have yet to be examined during a learning and memory task ^22,23^. Two particularly interesting candidate genes, *npas4* and *gabbr1 *(*npas4a* and *gabbr1a* in teleosts) are essential in regulating neuronal excitability and molecular processes related to learning and memory such as long-term potentiation^24–26^. *npas4* is an immediate early gene transcription factor that is predominantly expressed in the brain and enriched in the limbic regions. It is expressed through calcium signaling and is thought to induce primarily GABAergic inhibitory synapses in response to excitation and play an important role in homeostatic plasticity^25^. *gabbr1* codes for a metabotropic GABA B receptor, which has also been shown to play an important role reducing neuronal excitability through G-protein signaling-dependent slow, long lasting hyperpolarization of postsynaptic cells. Further, deletion or altered expression of both of these genes has been shown to cause abnormal synaptic plasticity, neurogenesis, and impaired learning and memory abilities^27–29^. Both of these genes were found to have significantly upregulated whole-brain expression at baseline in selectively-bred reactive zebrafish, which separately showed faster acquisition of a contextual conditioned fear response towards an aversive olfactory alarm cue (alarm substance)^22,30^. However, it is unknown if expression of these genes in specific brain regions are more directly associated with proactive–reactive differences in fear learning.

The basic neural substrates of fear learning have been well characterized, and are promising candidate sites where neural plasticity-related processes may regulate variation in fear learning capabilities. Traditionally, the basolateral amygdala is at the center of the fear system, with the hippocampus providing relevant associative information to allow for context-specific defensive responses fearful stimuli^31^. More recently other brain regions such as the bed nucleus of the stria terminalis (BNST), lateral septum (LS), and striatum have attracted greater interest due to their functional and structural connections with the hippocampal/amygdala affective forebrain, and their output to structures essential for behavioral and physiological responses to potential threats. The majority of this circuitry has been characterized in rodent models, with putatively homologous structures identified in the teleost forebrain which have also been shown to be critical for contextual fear learning and adaptive responses to stress^32–35^.

Here we used the high-stationary behavior (HSB; reactive) and low-stationary behavior (LSB; proactive) zebrafish strains to study the association between *npas4a* and *gabbr1a* expression and fear learning differences between proactive and reactive stress coping styles. Starting from wild-caught zebrafish, the HSB and LSB strains were generated and are maintained by artificial selection for opposing amounts of stationary behavior in the open field test^36^. Specifically, we bred fish together that spent at least 66.7% exhibiting stationary behavior (200 s out of a 300 s trial, fish did not exceed speed of 0.1 cm/s) during the trial period to produce a High Stationary Behavior (HSB) line and bred those spending at most 16.7% time displaying stationary behavior to produce a Low Stationary Behavior (LSB) line. Consistent with proactive (LSB) and reactive (HSB) stress coping styles, HSB fish show elevated behavioral and cortisol stress responses compared to LSB fish^36,46^. LSB fish developed larger caudal regions and faster swimming performance^37^. Whole-brain transcriptomic differences included genes primarily related to neurometabolism and neurotransmission^22,38–40^. Additionally, these divergent behavioral profiles between the strains are consistent across contexts and over time and have high repeatability^36,41,42^.

We trained fish from the HSB and LSB strains to associate alarm substance exposure with a context in one training trial, followed by a second assessment trial in the absence of the alarm substance. We then quantified *npas4a* and *gabbr1a* forebrain expression to investigate their potential link with differences in conditioned fear responses between alternative stress coping styles. We predict that an increased conditioned fear response in reactive zebrafish will be associated with increased expression of neural plasticity-related genes in the dorsal and medial portions of the dorsal telencephalon (Dm, Dl) and the dorsal, ventral, and supracomissural portions of the ventral telencephalon (Vd, Vv, Vs), putative homologues of the mammalian basolateral amygdala, hippocampus, striatum, lateral septum, and bed nucleus of the stria terminalis, respectively^32–35^.

## Methods

### Subjects

Zebrafish are utilized in a variety of laboratory studies to understand the neural, genetic, and pharmacological mechanisms of learning and memory^43–45^. Both wild and laboratory strains of zebrafish display the proactive and reactive stress coping styles, which have distinct genetic architectures and neuroendocrine responses^22,23,46^. Here we used the high-stationary behavior (HSB; reactive) and low-stationary behavior (LSB; proactive) zebrafish strains to study the association between *npas4a* and *gabbr1a* expression and fear learning differences between proactive and reactive stress coping styles. Starting from wild-caught zebrafish, the HSB and LSB strains were generated and are maintained by artificial selection for opposing amounts of stationary behavior to a novelty stressor^36^. Specifically, we bred fish together that spent at least 66.7% exhibiting stationary behavior (200 s out of a 300 s trial, fish did not exceed speed of 0.1 cm/s) during the trial period to produce a High Stationary Behavior (HSB) line and bred those spending at most 16.7% time displaying stationary behavior to produce a Low Stationary Behavior (LSB) line. The HSB and LSB strains show contrasting behavior, physiology, morphology, and neuromolecular profiles consistent with the reactive and proactive coping styles, respectively^22,36–40^. Additionally, these divergent behavioral profiles between the strains are consistent across contexts and over time and have high repeatability^36,41,42^. During testing, fish were individually housed in 3-L tanks on a recirculating water system (Pentair Aquatic Eco-Systems) using UV and solid filtration on a 14:10 L/D cycle at a temperature of 27 °C. Fish were fed twice a day with Tetramin Tropical Flakes (Tetra, USA). All procedures were approved by the Institutional Animal Care and Use Committee of University of Nebraska at Omaha/University of Nebraska Medical Center (17-070-00-FC, 17-064-08-FC). All experiments were performed in accordance with relevant guidelines and regulations.

### Alarm substance

We created a single batch of alarm substance as previously described^30^. In brief, 20 randomly selected donor fish (wild type) were euthanized by rapid chilling followed by light abrasion of lateral skin cells on one side of each donor fish, ensuring that no blood was drawn. Donor bodies were then individually soaked in 10 mL of DI water for 10 min. A total of 200 mL was filtered, diluted in half, and stored in aliquots at – 20 °C until use.

### Contextual fear learning

To test learning, we utilized a validated contextual fear conditioning paradigm^30^. Briefly, zebrafish were tested individually in a 16 × 16 × 10 cm arena filled with 1.4 L of system water. The arena was surrounded by opaque white plastic on the bottom and sides to serve as the contextual stimulus. Animals were removed from group housing and placed into individual housing 72 h prior to the training session. Each learning trial was 15 min long and was divided into three subsections. Fish acclimated to the chamber for the first five minutes, followed by five minutes of recording pre-exposure behavior (conditioned fear response for second trial). After these 10 min, 1 mL of alarm substance (AS) or distilled water (DI) was administered into the water through plastic tubing that came from outside of the testing arena. Following alarm substance exposure, the unconditioned fear response was recorded for five minutes. Between trials, fish were placed back into their individual housing, the testing arenas were rinsed out, and were refilled with 1.4 L of fresh system water. Fish underwent two training trials with 30 min between trials. The second training trial was stopped after the second five minute block (conditioned response). Fish immediately had their forebrains removed or were decapitated and frozen on dry ice and stored at − 80 °C for qPCR and ISH, respectively. We selected the second trial for gene expression analyses because we previously showed that out of four training trials, the second trial was both the earliest trial and one that resulted in the most prominent proactive–reactive behavioral differences during fear conditioning before both lines achieved similar conditioned responses. These differences during training were also associated with stronger fear memory recall 96 h following training^30^.

Total sample sizes consisted of 46 LSB (N = 28 males, 18 females) and 46 HSB (N = 28 males, 18 females) individuals. Of this total, we used 10 HSB individuals (N = 5 AS, 5 DI, all males) and 10 LSB individuals (N = 5 AS, 5 DI, all males) for qRT-PCR analysis. We used the remaining fish for ISH analysis. A total of 12 LSB (N = 6 males, 6 females) and 12 HSB (N = 6 males, 6 females) individuals received alarm substance CS-US reinforcements as the experimental group. For the DI water control group, we used 12 HSB (N = 6 males, 6 females) and 12 LSB (N = 6 males, 6 females) fish. To control for possible effects of the paradigm and handling, independent of treatment group, 12 HSB (N = 6 males, 6 females) and 12 LSB (N = 6 males, 6 females) were habituated to the same single housing as other groups, but did not undergo behavioral testing.

### Behavioral analysis

All trials were video-recorded from above and later analyzed with Noldus Ethovision XT (Noldus XT, Wageningen, Netherlands). For each trial, we quantified freezing time as an indicator of the conditioned response. We examined freezing because it is one of the most consistent and conserved behaviors used to assess stress-related behaviors and fear learning and memory^47^. Additionally, freezing was the most reliable indicator of proactive–reactive differences in contextual fear conditioning in our prior study^30^. The subject was considered frozen if it moved less than 0.5 cm/s.

### qRT-PCR

Preparation, execution, and analysis of the qRT-PCR of forebrain *npas4a* and *gabbr1a* expression followed previously established methods^38,39^. Gene expression was normalized to an endogenous housekeeping gene, *ef1a*, which has shown to be stable across sex, age, and chemical treatment in zebrafish^48^. See the supplemental methods for detailed parameters.

### ISH

Brain samples were sectioned on a cryostat at 16 µm onto four serial series. Tissue fixation parameters, probe synthesis, and ISH conditions were based on established protocols^49,50^. We used digoxigenin (DIG)-labeled probes for *Npas4a* and *Gabbr1a* genes. All individuals were processed simultaneously (one gene at a time) to avoid any potential colorimetric development differences across individuals due to batch effects. Riboprobes showed specific binding with high expression using the antisense probe, proportionally reduced expression in the 1:25 cold-competitor condition, and no expression in the sense and no probe conditions (Fig. S1). See supplemental methods for detailed parameters.

### Brain region analysis

Brain section images were captured at 4X using a Nikon Eclipse monochrome camera (Qi2). For each brain region, we used Nikon NIS Elements Version 4.6 software to measure a standardized rectangular box within the borders of each brain region and measured the mean intensity of *npas4a* and *gabbr1a* expression within the box. Brain regions were defined using a published teleost brain atlas and are displayed in Fig. S2^51^. The researcher (M.R.B.) was blinded to the treatment and strain conditions when collecting and analyzing images. We quantified gene expression by measuring optical density (OD) of the digoxigenin labeled probes, an established semi-quantitative measure of gene expression in other systems^49^. For each slide, we normalized the mean intensity of all measures to the background (mean intensity of slide area not containing tissue), which produced a fractional transmittance value for each brain region in each section. Fractional transmittance was mathematically converted to optical density by the equation OD = 2 − log(fractional transmittance). See supplemental methods for additional details.

### Statistics

All statistics were performed using SPSS software (Version 24). To analyze freezing behavior we used a repeated measures two-way ANOVA with strain and treatment group as between-subjects factors. For analyzing qRT-PCR gene expression we used a multivariate general linear model (GLM) with normalized *npas4a* and *gabbr1a* expression as dependent variables, and strain and treatment as between-subject factors. For analysis of ISH OD measurements we used a multivariate GLM with the OD of the five brain regions as dependent variables and strain and treatment group as between-subjects factors. There were not any effects of sex on learning and memory in a previous nor the current study (3-way repeated measures ANOVA: F_sex × trial_ = 0.40 p = 0.531; F_sex_ = 0.57 p = 0.456), so we removed sex as a variable to simplify the model^30^. Individual groups were compared with simple effects testing. To account for multiple comparisons we applied the Benjamini–Hochberg correction to determine significance^52^. For all significant differences (p < 0.05) we also report the effect sizes (Cohen’s d (d) for t-tests and partial eta-squared (ηp^2^) for ANOVAs^53^. All effect sizes were medium or large effects^53–55^.

### Ethical approval

The manuscript has been reviewed and approved by all listed authors for publication. All procedures were approved by the Institutional Animal Care and Use Committee of University of Nebraska at Omaha/University of Nebraska Medical Center (17-070-00-FC, 17-064-08-FC).

## Results

### Contextual fear learning

**Figure 1:**
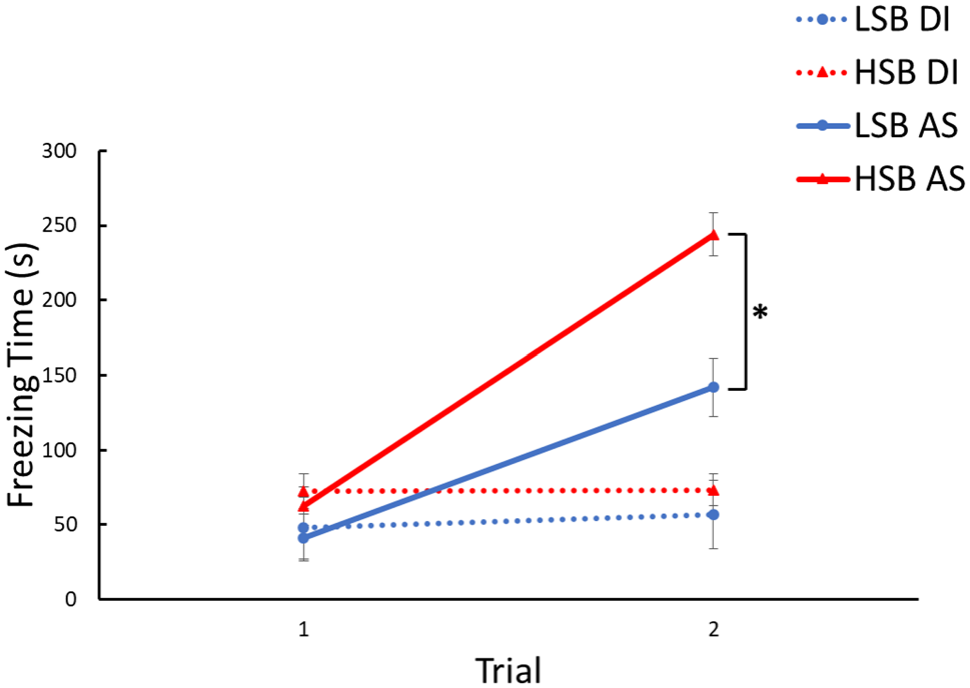
Acquisition of fear memory over two training trials. Freezing time during the conditioned freezing period was measured for high stationary behavior (HSB) and low stationary behavior (LSB) fish exposed to distilled water (DI) or alarm substance (AS). Points represent mean ± 1 standard error. × Indicates *p* < 0.05 for within-treatment group comparison.

In the conditioned fear response period during acquisition testing, there was a significant trial × treatment group interaction effect for freezing (*F*_1, 64_ = 54.86, *p* = 3.59 × 10^–10^, ηp^2^ = 0.46). The alarm substance group showed increased freezing between trials at a faster rate than the DI control group (Fig. 1). Additionally, there was a significant trial × strain × treatment group interaction (*F*_1, 64_ = 5.88, *p* = 0.018, ηp^2^ = 0.08) where treated HSB fish increased freezing behavior at a faster rate than LSB fish. HSB fish exposed to alarm substance froze significantly more than LSB fish at trial two (*t*(32) = 4.23, *p* = 1.81 × 10^–4^, d = 1.45), but did not significantly differ at trial one (*t*(32) = 1.05, *p* = 0.303). Full model results are presented in Table 1.

**Table 1:** Results of repeated measures GLM for the acquisition learning phase for freezing time.

	Freezing time F(p, ηp^2^)
**Within-subjects effects (df = 1, 64)**	
Trial	**62.82 (4.36 × 10**^**–11**^**, 0.50)**
Trial × strain	3.89 (0.053)
Trial × treatment	**54.86 (3.59 × 10**^**–10**^**, 0.46)**
Trial × strain × treatment	**5.88 (0.018, 0.08)**
**Between subjects effects (df = 1, 64)**	
Intercept	**179.53 (3.08 × 10**^**–20**^**, 0.74)**
Strain	**8.92 (0.004, 0.12)**
Treatment	**18.78 (5.30 × 10**^**–5**^**, 0.23)**
Strain × treatment	2.18 (0.144)

Bold text indicates *p* < 0.05.

### qRT-PCR

There was a significant effect of strain on both *npas4a* (*F*_1, 16_ = 11.72, *p* = 0.003, ηp^2^ = 0.42) and *gabbr1a* (*F*_1, 16_ = 7.29, *p* = 0.016, ηp^2^ = 0.31) forebrain expression. There was a significant effect of treatment for *npas4a* (*F*_1, 16_ = 11.72, *p* = 0.003, ηp^2^ = 0.42), but not *gabbr1a* (*F*_1, 16_ = 4.30, *p* = 0.055) expression. Full model results are presented in Table 2. In HSB fish, *npas4a* gene expression was significantly higher in the AS group compared to the DI group (*p* = 0.003, d = 2.34; Fig. S3). There were no effects of treatment on *npas4a* expression in LSB fish (p = 0.918).

**Table 2:** Results of multivariate GLM for forebrain expression of *npas4a* and *gabbr1a* from qPCR.

	*npas4a* F (p, ηp^2^)	*gabbr1a* F (p, ηp^2^)
Intercept	**393.93 (1.08 × 10**^**–12**^**, 0.96)**	**364.98 (1.94 × 10**^**–12**^**, 0.96)**
Strain	**11.72 (0.003, 0.42)**	**7.29 (0.016, 0.31)**
Treatment	**11.72 (0.003, 0.42)**	4.30 (0.055)
Strain × Treatment	2.32 (0.147)	3.88 (0.066)

Bold text indicates *p* < 0.05.

### In situ hybridization

#### Treatment effects on npas4a OD

**Figure 2:**
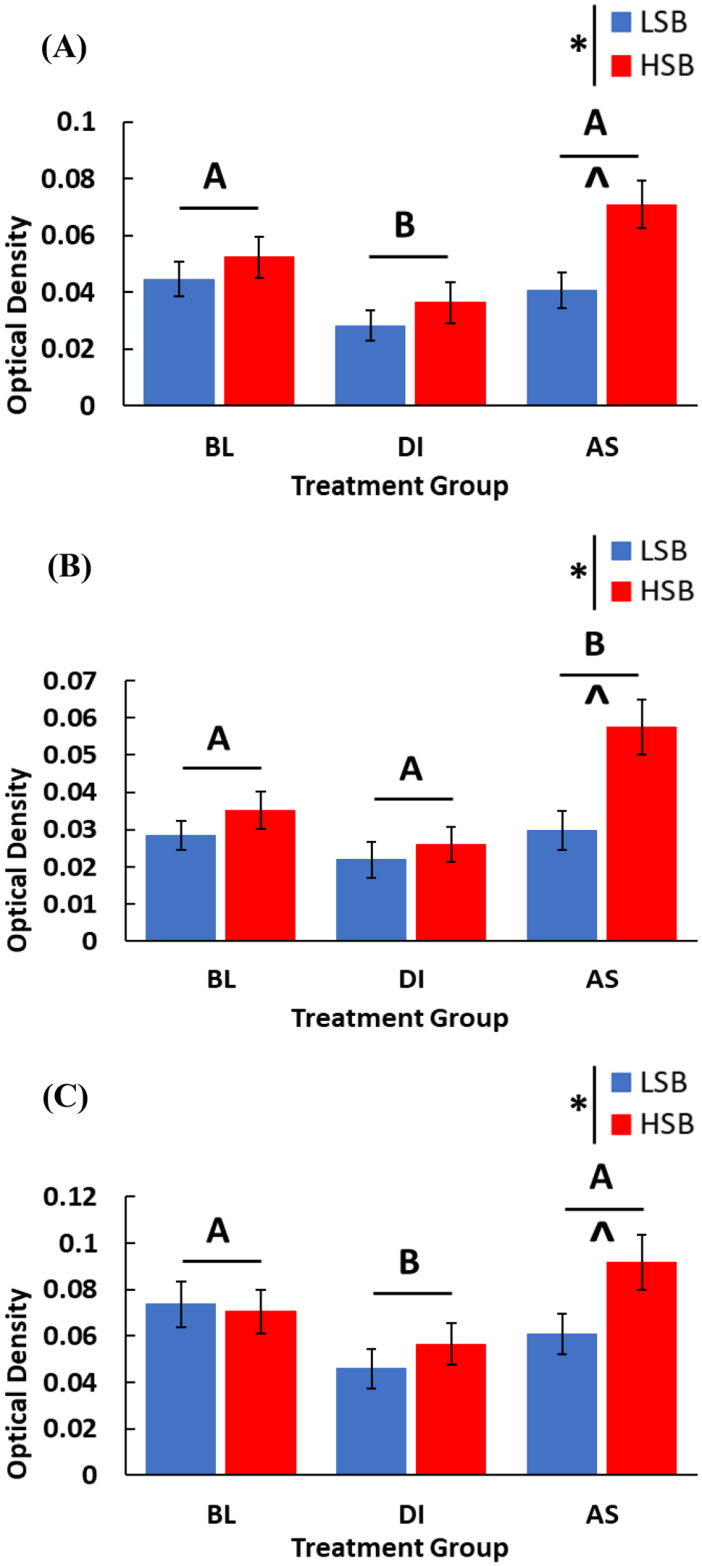
Expression of *npas4a* in the Dm (**A**), Dl (**B**), and Vs (**C**). We measured expression of high stationary behavior (HSB) and low stationary behavior (LSB) fish at baseline (BL) or exposed to either alarm substance (AS) or distilled water (DI) during training. Bars represent mean ± 1 SE. Bars labeled with different letters indicate p < 0.05. × Indicates a significant strain main effect. ^Indicates a significant within-treatment group strain difference.

There was a significant effect of treatment condition on *npas4a* OD in the Dm (*F*_2, 66_ = 6.20, *p* = 0.003, ηp^2^ = 0.16), Dl (*F*_2, 66_ = 7.13, *p* = 0.002, ηp^2^ = 0.18), Vv (*F*_2, 66_ = 3.38, *p* = 0.040, ηp^2^ = 0.09), and Vs (*F*_2, 66_ = 3.93, *p* = 0.024, ηp^2^ = 0.11). In the Dm, *npas4a* OD was significantly lower in DI water treatment group compared to both the baseline (*p* = 0.030, d = 0.67) and alarm substance group (*p* = 0.003, d = 1.04; Fig. 2A). In the Dl, *npas4a* OD was significantly higher in the AS group compared to both the baseline (*p* = 0.042, d = 0.63) and DI water treatment group (*p* = 0.003, d = 1.05; Fig. 2B). In the Vv, the AS group initially had a significantly higher OD compared to the baseline (*p* = 0.048, d = 0.59) and DI groups (*p* = 0.018, d = 0.71), however this was not significant after BH correction (*p* = 0.072, 0.054 respectively; Fig. S4A). In the Vs, *npas4a* OD was significantly lower in the DI group compared to both the baseline (*p* = 0.039, d = 0.62) and AS treatment group (*p* = 0.033, d = 0.74; Fig. 2C). In the Vd, *npas4a* OD was significantly higher in the AS group compared to the DI group for LSB fish only (*p* = 0.002, d = 1.00; Fig. S4B).

#### Strain effects on Npas4a OD

**Figure 3:**
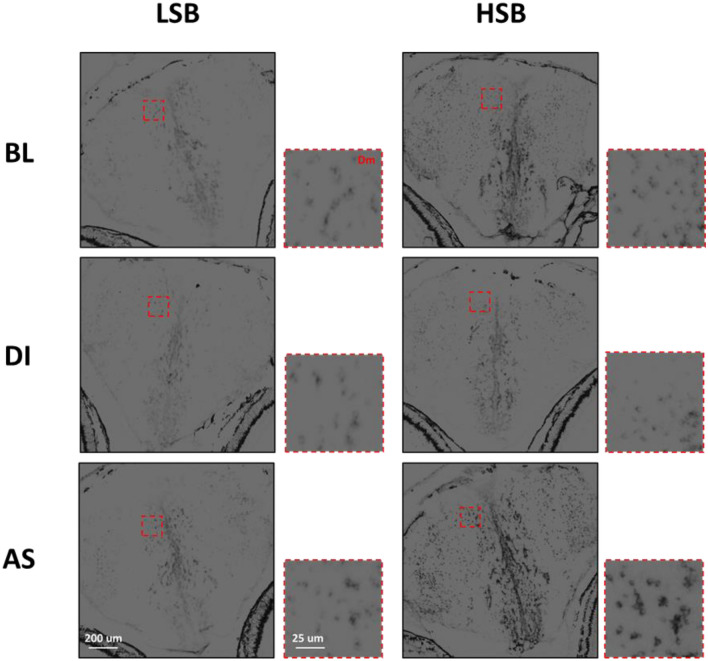
Representative images of *Npas4a* expression in the Dm for low stationary behavior (LSB) and high stationary behavior (HSB) fish at baseline (BL) or exposed to either alarm substance (AS) or distilled water (DI) during training.

**Figure 4:**
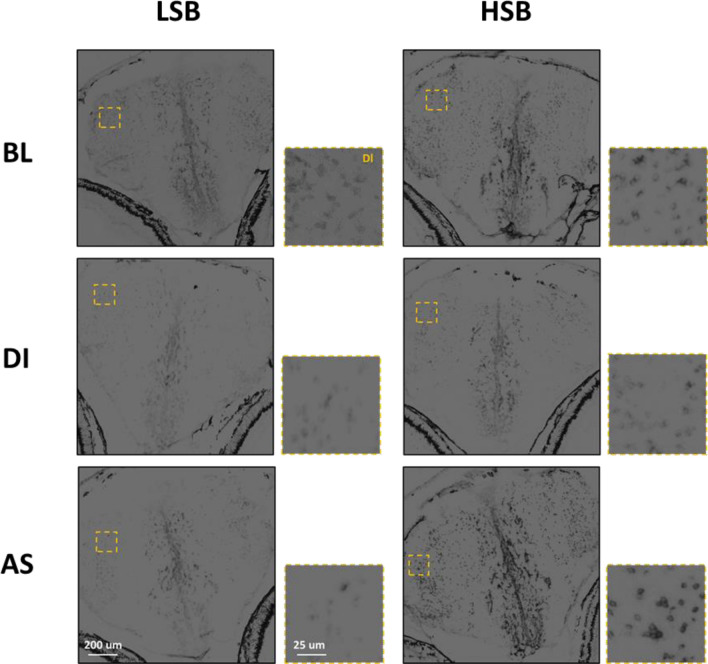
Representative images of *Npas4a* expression in the Dl for low stationary behavior (LSB) and high stationary behavior (HSB) fish at baseline (BL) or exposed to either alarm substance (AS) or distilled water (DI) during training.

**Figure 5:**
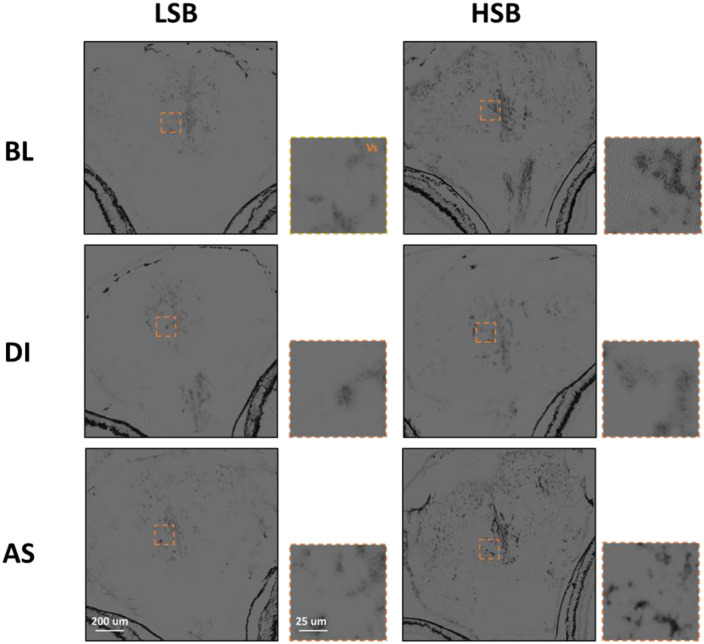
Representative images of *Npas4a* expression in the Vs for low stationary behavior (LSB) and high stationary behavior (HSB) fish at baseline (BL) or exposed to either alarm substance (AS) or distilled water (DI) during training.

There was a significant main effect of strain on the OD of *npas4a* in the Dm (*F*_1, 66_ = 7.66, *p* = 0.007, ηp^2^ = 0.10), Dl (*F*_1, 66_ = 8.82, *p* = 0.004, ηp^2^ = 0.12), and Vv (*F*_1, 66_ = 5.16, *p* = 0.026, ηp^2^ = 0.07). HSB fish overall had higher OD of *npas4a* in each of the three brain regions. Additionally, HSB fish exposed to AS had significantly higher *npas4a* OD compared to LSB fish exposed to AS in the Dm (*p* = 0.001, d = 1.25), Dl (*p* = 0.001, d = 1.65), and Vs (*p* = 0.039, d = 0.65; Fig. 2A–C). Full model results are presented in Table 3. Representative raw images for *npas4a* expression in the Dm, Dl, and Vs are shown in Figs. 3, 4, and 5. Npas4a expression in the Vv and Vd are shown in Figs. S5–S6.

**Table 3:** Results of multivariate GLM of *npas4a* optical density across the five forebrain regions.

	Dm	Dl	Vv	Vd	Vs
	F(p, ηp^2^)	F(p, ηp^2^)	F(p, ηp^2^)	F(p, ηp^2^)	F(p, ηp^2^)
Intercept	**266.15 (4.36 × 10**^**–4**^** , 0.80)**	**236.22 (4.36 × 10**^**–4**^**, 0.78)**	**295.70 (4.36 × 10**^**–4**^**, 0.82)**	**282.1 (4.36 × 10**^**–4**^**, 0.81)**	**286.57 (4.36 × 10**^**–4**^**, 0.81)**
Strain	**7.66 (0.007, 0.10)**	**8.82 (0.004, 0.12)**	**5.16 (0.026, 0.07)**	0.77 (0.383)	2.64 (0.109)
Treatment	**6.20 (0.003, 0.16)**	**7.13 (0.002, 0.18)**	**3.38 (0.040, 0.09)**	1.61 (0.208)	**3.93 (0.024, 0.11)**
Strain × Treatment	1.78 (0.177)	3.02 (0.055)	0.91 (0.406)	**4.51 (0.015, 0.12)**	1.59 (0.212)

Bold text indicates *p* < 0.05.

#### Strain specific treatment effects on gabbr1a OD

For *gabbr1a* OD, there were significant strain × treatment group interaction effects in the Dm (*F*_1, 66_ = 3.31, *p* = 0.043, ηp^2^ = 0.09), Vv (*F*_1, 66_ = 7.70, *p* = 0.001, ηp^2^ = 0.19), Vd (*F*_1, 66_ = 6.95, *p* = 0.002, ηp^2^ = 0.17), and Vs (*F*_1, 66_ = 3.89, *p* = 0.025, ηp^2^ = 0.11). For each of those regions, there were no significant differences between any treatment groups for HSB fish. However, for LSB fish the DI group had significantly lower *gabbr1a* OD compared to the BL (*p* = 0.003, d = 1.37) and AS (*p* = 0.024, d = 1.00) groups in the Dm, BL (*p* = 0.023, d = 1.02) and AS (*p* = 0.003, d = 1.60) groups in the Vv, and the BL (*p* = 0.015, d = 1.06) and AS (*p* = 0.003, d = 1.37) groups in the Vd (Fig. S7). The BL group had a significantly higher *gabbr1a* OD compared to the DI (*p* = 0.003, d = 1.71) and AS (*p* = 0.030, d = 0.99) groups in the Vs. Full model results are presented in Table 4. Representative raw images for *gabbr1a* expression across all of the brain regions and treatment groups are shown in Figs. S8–S12.

**Table 4:** Results of multivariate GLM of gabbr1a optical density across the five forebrain regions.

	Dm	Dl	Vv	Vd	Vs
	F(p, ηp^2^)	F(p, ηp^2^)	F(p, ηp^2^)	F(p, ηp^2^)	F(p, ηp^2^)
Intercept	**121.69** **(1.27 × 10**^**–16**^**, 0.65)**	**107.89** **(1.61 × 10**^**–15**^**, 0.62)**	**147.75** **(1.68 × 10**^**–18**^**, 0.69)**	**153.60** **(6.87 × 10**^**–19**^**, 0.70)**	**134.16** **(1.49 × 10**^**–17**^**, 0.67)**
Strain	0.44 (0.509)	2.91 (0.093)	0.166 (0.685)	0.59 (0.444)	0.12 (0.736)
Treatment	**3.28 (0.044, 0.09)**	2.69 (0.076)	1.52 (0.227)	0.91 (0.410)	**5.88 (0.004, 0.15)**
Strain × treatment	**3.31 (0.043, 0.09)**	1.51 (0.229)	**7.70 (.001, .19)**	**6.95 (0.002, 0.17)**	**3.89 (0.025, 0.11)**

Bold text indicates *p* < 0.05.

## Discussion

Expression of neural plasticity-related genes (e.g. *npas4, gabbr1a*) has been broadly implicated as a key process underlying alternative stress coping styles, but has not been investigated related to proactive–reactive differences in learning and memory^20–22,26,27,56,57^. Consistent with previous findings, we found that reactive (HSB) zebrafish showed an increased conditioned fear response relative to proactive (LSB) individuals (Fig. 1)^30^. Further, we found that *npas4a* expression was significantly higher in several key forebrain regions of reactive zebrafish. Altogether, our findings suggest that *npas4a* plays a similar role in learning and memory as its mammalian homolog, and may be an important regulator of proactive–reactive differences in learning and memory.

ISH analysis showed that *npas4a* expression was significantly higher in reactive fish in the Dm, Dl, and Vs (Fig. 2A–C). The Dm (BLA), Dl (hippocampus), and Vs (BNST) are key sites of experience-dependent plasticity and integral to fear learning and memory across species^32–35^. Similar to rodents, lesioning the teleost Dm and Dl impairs the formation of new fear and contextual memories^33,58–60^. Our findings suggest that *npas4a*-dependent plasticity within these brain regions may be a key underlying mechanism regulating differences in fear learning and memory capabilities between stress coping styles. In a prior study using the same conditioning paradigm, we showed that reactive zebrafish acquired a conditioned fear response faster than proactive zebrafish^30^. The higher activity-dependent expression of *npas4a* in reactive individuals observed in this study may promote higher levels of neural plasticity, resulting in salient and fearful experiences to be encoded into memory more quickly^28,61^. We predict that *npas4a* knockout experiments would produce similar learning and memory deficits as in rodents, and are needed to establish a direct causal role in zebrafish. More recently, specific glutamatergic populations of Dm cells have been shown to be required for fear conditioning^32^. Our study is not able to distinguish between cell types expressing *npas4a*, which would be an important future direction to better characterize the specific circuits regulating proactive–reactive differences in learning. In selectively bred proactive and reactive trout, the Dm and Dl regions have also been shown to display differing monoaminergic and cortisol responses to acute stress^62,63^. This suggests that higher expression of *npas4a* in these brain regions may play important roles in constraining variation across a number of behavioral contexts.

While the BNST has been shown to be important for aversive learning in rodents^64,65^, the function of the Vs and specifically of *npas4a* expression in the Vs is not well understood in regards to learning and memory. We found that similar to the Dm and Dl, *npas4a* expression within the Vs is likely important for fear learning, and is associated with differences between proactive and reactive stress coping styles. Supporting this, a previous study found that increased activity and *npas4* expression in a population of corticotropin-releasing factor neurons in the BNST was associated with increased stress resiliency and prevention of a post-traumatic stress disorder-like phenotype in rodents^66^. This suggests that *npas4a* expression in the Vs may play an important role in how individuals experience and cope with stress differently. Interestingly, the Vs has been shown to have connections with both the Dm and Dl, and to the hypothalamus and other brainstem areas that are essential for eliciting behavioral and endocrine stress responses^67^. While this study only assessed gene expression across select forebrain structures, future studies should investigate other downstream structures and consider the role of glucocorticoids and the hypothalamus–pituitary–adrenal axis (hypothalamus-pituitary-interrenal in teleosts). This is particularly promising as glucocorticoid differences have been well-characterized between proactive and reactive stress coping styles^3,68–70^, though to a lesser extent related to learning and memory.

The DI treatment groups showed significantly lower *npas4a* expression compared to the AS treatment group in the Dm, Dl, and Vs (Fig. 2a,c). This suggests *npas4*a is expressed in a treatment-specific manner associated with the learned conditioned fear response in the AS group. Unexpectedly, *npas4a* expression in the DI group was significantly lower than the BL group in the Dm and Vs. Other studies have found that acute injection of corticosterone or chronic restraint and social isolation stressors can decrease *npas4* expression in the rodent prefrontal cortex and hippocampus and lead to a variety of behavioral deficits including learning and memory^71–73^. It is unclear whether this decrease in expression is maladaptive, or whether it is an adaptive homeostatic response to stress^74^. It is unlikely that our results can be explained by physical isolation, as the baseline group was also socially isolated for the same duration. However, it is possible that handling stress could explain the reduction in *npas4a* expression for the DI group.

It is important to note that the relationship between *npas4a* expression and behavior could also represent stress coping style differences to an acute stress response, given that the assessment trial for conditioned fear response is roughly 30 min following the first exposure. Some studies have shown differences in freezing post-alarm substance exposure^36^, though we did not find any unconditioned fear response differences with the current paradigm. While this could partially explain our findings, our previous study using similar repeated alarm substance exposure showed that proactive and reactive zebrafish developed a conditioned fear memory that was context-specific and persisted for at least 4 days^30^.

While qRT-PCR findings showed strain effects in *gabbr1a* expression, there were no strain differences in any of the analyzed brain regions for the ISH analysis. This suggests that the strain differences in forebrain *gabbr1a* expression are driven by other brain regions not investigated in this study. Therefore, *gabbr1a* expression within the Dm, Dl, Vv, Vs, and Vd does not appear to be associated with development of a conditioned fear response. Other studies have suggested that GABAergic signaling may be more important for consolidation, reconsolidation, or extinction of fear memories^75^. Future studies should assess how GABA B receptor expression may influence other phases of fear conditioning, or other paradigms using positive reinforcement.

Learning to predict and cope with potentially dangerous environments is essential to an individual’s survival. Proactive and reactive stress coping styles represent alternative strategies for coping with stress and differ in a number of behavioral contexts, including learning and memory. Our study suggests that brain-region specific expression patterns of *npas4a* may underlie differences in fear learning between proactive and reactive stress coping styles. These findings advance our understanding of the neuromolecular mechanisms underlying stress-coping style differences in cognition and highlight neuroplasticity’s key role in regulating alternative adaptive behavioral responses to stress. Additionally, as proactive and reactive individuals share potentially conserved mechanisms underlying other stress coping behaviors, this suggests that these brain regions may also constrain behavioral variation in a number of disparate contexts.

## Supplementary Information

Supplementary Information.

## References

[1] Baker MR, Hofmann HA, Wong RY (2001). Neurogenomics of Behavioural Plasticity in Socioecological Contexts.

[2] Koolhaas JM (1999). Coping styles in animals: Current status in behavior and stress-physiology. Neurosci. Biobehav. Rev..

[3] Koolhaas JM, de Boer SF, Coppens CM, Buwalda B (2010). Neuroendocrinology of coping styles: Towards understanding the biology of individual variation. Front. Neuroendocrinol..

[4] Øverli Ø (2007). Evolutionary background for stress-coping styles: Relationships between physiological, behavioral, and cognitive traits in non-mammalian vertebrates. Neurosci. Biobehav. Rev..

[5] Brown GE (2013). Retention of acquired predator recognition among shy versus bold juvenile rainbow trout. Behav. Ecol. Sociobiol..

[6] Dougherty LR, Guillette LM (2018). Linking personality and cognition: a meta-analysis. Philos. Trans. R. Soc. B. Biol. Sci..

[7] Lucon-Xiccato T, Bisazza A (2017). Individual differences in cognition among teleost fishes. Behav. Process..

[8] Miller N (2017). Cognition in fishes. Behav. Process..

[9] Sih A, Del Giudice M (2012). Linking behavioural syndromes and cognition: A behavioural ecology perspective. Philos. Trans. R. Soc. B. Biol. Sci..

[10] Amy M, van Oers K, Naguib M (2012). Worms under cover: Relationships between performance in learning tasks and personality in great tits (*Parus major*). Anim. Cogn..

[11] Bolhuis JE, Schouten WGP, De LJA, Schrama JW, Wiegant VM (2004). Individual coping characteristics, rearing conditions and behavioural flexibility in pigs. Behav. Brain Res..

[12] DePasquale C, Wagner T, Archard GA, Ferguson B, Braithwaite VA (2014). Learning rate and temperament in a high predation risk environment. Oecologia.

[13] Dugatkin LA, Alfieri MS (2003). Boldness, behavioral inhibition and learning. Ethol. Ecol. Evol..

[14] Mazza V, Eccard JA, Zaccaroni M, Jacob J, Dammhahn M (2018). The fast and the flexible: Cognitive style drives individual variation in cognition in a small mammal. Anim. Behav..

[15] Mesquita FO, Borcato FL, Huntingford FA (2015). Cue-based and algorithmic learning in common carp: A possible link to stress coping style. Behav. Process..

[16] Trompf L, Brown C (2014). Personality affects learning and trade-offs between private and social information in guppies, *Poecilia reticulata*. Anim. Behav..

[17] Budaev SV, Zhuikov AY (1998). Avoidance learning and “personality” in the guppy (*Poecilia**reticulata*). J. Comp. Psychol..

[18] Exnerová A, Svádová KH, Fučíková E, Drent P, Štys P (2010). Personality matters: Individual variation in reactions of naive bird predators to aposematic prey. Proc. R. Soc. B. Biol. Sci..

[19] Miller KA, Garner JP, Mench JA (2006). Is fearfulness a trait that can be measured with behavioural tests? A validation of four fear tests for Japanese quail. Anim Behav..

[20] Øverli Ø, Sørensen C (2016). On the role of neurogenesis and neural plasticity in the evolution of animal personalities and stress coping styles. Brain Behav. Evol..

[21] Sørensen C, Johansen IB, Øverli Ø (2013). Neural plasticity and stress coping in teleost fishes. Gen. Comp. Endocrinol..

[22] Wong RY, Lamm MS, Godwin J (2015). Characterizing the neurotranscriptomic states in alternative stress coping styles. BMC Genom..

[23] Oswald ME, Singer M, Robison BD (2013). The quantitative genetic architecture of the bold-shy continuum in zebrafish, *Denio rerio*. PLoS ONE.

[24] Kfir A (2014). Learning-induced modulation of the GABAB-mediated inhibitory synaptic transmission: Mechanisms and functional significance. J.. Neurophysiol..

[25] Lin Y (2008). Activity-dependent regulation of inhibitory synapse development by Npas4. Nature.

[26] Maya-Vetencourt JF (2012). Experience-dependent expression of NPAS4 regulates plasticity in adult visual cortex. J. Physiol..

[27] Heaney CF, Kinney JW (2016). Role of GABABreceptors in learning and memory and neurological disorders. Neurosci. Biobehav. Rev..

[28] Ploski JE, Monsey MS, Nguyen T, DiLeone RJ, Schafe GE (2011). The neuronal PAS domain protein 4 (Npas4) is required for new and reactivated fear memories. PLoS ONE.

[29] Ramamoorthi K (2011). Npas4 regulates a transcriptional program in CA3 required for contextual memory formation. Science.

[30] Baker MR, Wong RY (2019). Contextual fear learning and memory differ between stress coping styles in zebrafish. Sci. Rep..

[31] Maren S, Phan KL, Liberzon I (2013). The contextual brain: Implications for fear conditioning, extinction and psychopathology. Nat. Rev. Neurosci..

[32] Lal P (2018). Identification of a neuronal population in the telencephalon essential for fear conditioning in zebrafish. BMC Biol..

[33] Ganz J (2015). Subdivisions of the adult zebrafish pallium based on molecular marker analysis. F1000 Res..

[34] de CarmoSilva RX, Lima-Maximino MG, Maximino C (2018). The aversive brain system of teleosts: Implications for neuroscience and biological psychiatry. Neurosci. Biobehav. Rev..

[35] Panula P (2010). The comparative neuroanatomy and neurochemistry of zebrafish CNS systems of relevance to human neuropsychiatric diseases. Neurobiol. Dis..

[36] Wong RY (2012). Comparing behavioral responses across multiple assays of stress and anxiety in zebrafish (*Danio rerio*). Behaviour.

[37] Kern EMA, Robinson D, Gass E, Godwin J, Langerhans RB (2016). Correlated evolution of personality, morphology and performance. Anim. Behav..

[38] Wong RY, McLeod MM, Godwin J (2014). Limited sex-biased neural gene expression patterns across strains in Zebrafish (*Danio rerio*). BMC Genom..

[39] Wong RY, Oxendine SE, Godwin J (2013). Behavioral and neurogenomic transcriptome changes in wild-derived zebrafish with fluoxetine treatment. BMC Genom..

[40] Goodman AC, Wong RY (2020). Differential effects of ethanol on behavior and GABAA receptor expression in adult zebrafish (*Danio rerio*) with alternative stress coping styles. Sci. Rep..

[41] Baker MR, Goodman AC, Santo JB, Wong RY (2018). Repeatability and reliability of exploratory behavior in proactive and reactive zebrafish *Danio rerio*. Sci. Rep..

[42] Johnson ZV (2020). Exploratory behaviour is associated with microhabitat and evolutionary radiation in Lake Malawi cichlids. Anim. Behav..

[43] Gerlai R (2016). Learning and memory in zebrafish (*Danio rerio*). Methods Cell Biol..

[44] Norton W, Bally-Cuif L (2010). Adult zebrafish as a model organism for behavioural genetics. BMC Neurosci..

[45] Oliveira RF (2013). Mind the fish: Zebrafish as a model in cognitive social neuroscience. Front. Neural Circuits.

[46] Wong RY, French J, Russ JB (2019). Differences in stress reactivity between zebrafish with alternative stress coping styles. R. Soc. Open Sci..

[47] Maren S (2001). Neurobiology of Pavlovian fear conditioning. Annu. Rev. Neurosci..

[48] McCurley AT, Callard GV (2008). Characterization of housekeeping genes in zebrafish: Male-female differences and effects of tissue type, developmental stage and chemical treatment. BMC Mol. Biol..

[49] Wong RY, Ramsey ME, Cummings ME (2012). Localizing brain regions associated with female mate preference behavior in a swordtail. PLoS ONE.

[50] Wong RY, Cummings ME (2014). Expression patterns of Neuroligin-3 and tyrosine hydroxylase across the brain in mate choice contexts in female swordtails. Brain Behav Evol.

[51] Wullimann MF, Rupp B, Reichert H (1996). Neuroanatomy of the zebrafish brain: A topological. Atlas.

[52] Benjamini Y, Drai D, Elmer G, Kafkafi N, Golani I (2001). Controlling the false discovery rate in behavior genetics research. Behav. Brain Res..

[53] Wassertheil S, Cohen J (1970). Statistical power analysis for the behavioral sciences. Biometrics.

[54] Starkings S (2012). IBM SPSS statistics 19 made simple by Colin D. Gray and Paul R. Kinnear. Int. Stat. Rev..

[55] Richardson JTE (2011). Eta squared and partial eta squared as measures of effect size in educational research. Educ. Res. Rev..

[56] Benito E, Barco A (2015). The neuronal activity-driven transcriptome. Mol. Neurobiol..

[57] Vertkin I (2015). GABA _B_ receptor deficiency causes failure of neuronal homeostasis in hippocampal networks. Proc. Natl. Acad. Sci..

[58] von Trotha JW, Vernier P, Bally-Cuif L (2014). Emotions and motivated behavior converge on an amygdala-like structure in the zebrafish. Eur. J. Neurosci..

[59] Ganz J (2012). Subdivisions of the adult zebrafish subpallium by molecular marker analysis. J. Comp. Neurol..

[60] Perathoner S, Cordero-Maldonado ML, Crawford AD (2016). Potential of zebrafish as a model for exploring the role of the amygdala in emotional memory and motivational behavior. J. Neurosci. Res..

[61] Qiu J (2016). Decreased Npas4 and Arc mRNA levels in the hippocampus of aged memory-impaired wild-type but not memory preserved 11β-HSD1 deficient mice. J. Neuroendocrinol..

[62] Vindas MA (2017). How do individuals cope with stress? Behavioural, physiological and neuronal differences between proactive and reactive coping styles in fish. J. Exp. Biol..

[63] Øverli Ø, Pottinger TG, Carrick TR, Øverli E, Winberg S (2001). Brain monoaminergic activity in rainbow trout selected for high and low stress responsiveness. Brain. Behav. Evol..

[64] Walker DL, Toufexis DJ, Davis M (2003). Role of the bed nucleus of the stria terminalis versus the amygdala in fear, stress, and anxiety. Eur. J. Pharmacol..

[65] Goode TD, Maren S (2017). Role of the bed nucleus of the stria terminalis in aversive learning and memory. Learn. Mem..

[66] Henckens MJAG (2017). CRF receptor type 2 neurons in the posterior bed nucleus of the stria terminalis critically contribute to stress recovery. Mol. Psychiatry.

[67] Rink E, Wullimann MF (2004). Connections of the ventral telencephalon (subpallium) in the zebrafish (*Danio rerio*). Brain Res..

[68] Boulton K (2015). How integrated are behavioral and endocrine stress response traits? A repeated measures approach to testing the stress-coping style model. Ecol. Evol..

[69] Baugh AT (2012). Corticosterone responses differ between lines of great tits (*Parus major*) selected for divergent personalities. Gen. Comp. Endocrinol..

[70] Wong RY, French J, Russ JB (2018) Differences in stress reactivity between zebrafish with alternative stress coping styles. Dissertation (University of Nebraska at Omaha). 10.1098/rsos.181797 PMC6549991 31218026

[71] Furukawa-Hibi Y, Yun J, Nagai T, Yamada K (2012). Transcriptional suppression of the neuronal PAS domain 4 (Npas4) gene by stress via the binding of agonist-bound glucocorticoid receptor to its promoter. J. Neurochem..

[72] Ibi D (2008). Social isolation rearing-induced impairment of the hippocampal neurogenesis is associated with deficits in spatial memory and emotion-related behaviors in juvenile mice. J. Neurochem..

[73] Yun J (2010). Chronic restraint stress impairs neurogenesis and hippocampus-dependent fear memory in mice: Possible involvement of a brain-specific transcription factor Npas4. J. Neurochem..

[74] Sun X, Lin Y (2016). Npas4: Linking neuronal activity to memory. Trends Neurosci..

[75] Makkar SR, Zhang SQ, Cranney J (2010). Behavioral and neural analysis of GABA in the acquisition, consolidation, reconsolidation, and extinction of fear memory. Neuropsychopharmacology.

