# Supplementary material for: Npas4a expression in the teleost forebrain is associated with stress coping style differences in fear learning
Source: Sci Rep. 2021 Jun 8;11:12074. doi: 10.1038/s41598-021-91495-7 (PMC8187387; doi:10.1038/s41598-021-91495-7)
Supplement: Supplementary file 1 — Supplementary Information. [file 41598_2021_91495_MOESM1_ESM.docx]

Title: *Npas4a* expression in the teleost forebrain is associated with stress coping style differences in fear learning

Matthew R Baker^a^ and Ryan Y Wong^a,b*^

^a^ Department of Biology, University of Nebraska at Omaha

^b^ Department of Psychology, University of Nebraska at Omaha

*Correspondence: Ryan Y Wong, University of Nebraska at Omaha, 6001 Dodge St, Omaha, NE 68182 Email: [rwong@unomaha.edu](mailto:rwong@unomaha.edu) Phone: 402-554-4473

**Supplementary Information**

**Methods**

*qRT-PCR*

We homogenized the tissue in Tri reagent (Sigma) and zirconium oxide beads in a Bullet Blender (NextAdvance) and extracted the RNA through column filtration (RNeasy Micro Plus Kit, Qiagen). RNA was subsequently converted to cDNA (Superscript IV First-Strand Synthesis System, Invitrogen) and purified (Millipore Amicon Ultra -0.5 mL 30 K Centrifugal Filters Devices). We ran qRT-PCR reactions on a QuantStudio 7 Flex Real-Time PCR system (Applied Biosystems) using PowerUp SYBR Green Master Mix (Applied Biosystems). A 131 base pair *npas4a* amplicon was created using 5’-CACCTCGGACACTCAATGGT-3’ (F) and 5’-AACAAGCGATCTGTGTCAGGT-3’ (R) as primers. A 198 base pair *gabbr1a* amplicon was created using 5’-CCCAGAGACGGAGGGATACG-3’ (F) and 5’-CGGGCACATCATCAAGCATCT-3’ (R) as primers. The parameters for both genes were as follows: 2 minutes at 50^o^C, 2 minutes at 95^o^C, followed by 40 cycles of 15 seconds of 95^o^C and 1 minute of 60^o^C. Primer concentration was 5 pmole/µl for both genes.

*Tissue Section Processing*

All series were simultaneously post-fixed in cold 4% paraformaldehyde/PBS solution, washed in PBS and acetylated in 0.25% acetic anhydride/triethanolamine. Then, slides were washed in 2X standard saline citrate, dehydrated in increasing ethanol series and stored at -80 ^o^C.

*Probe Synthesis*

To quantify *npas4a* and *gabbr1a* we used digoxigenin (DIG)-labeled RNA probes. A 402 base pair *npas4a* DIG probe template was subcloned by using primer pair 5’- TTCTGTAGCGTCCAATCGGC -3’and 5’- ACTTCCACTCCCATCTTTGCG -3’. The 390 base pair *gabbr1a* probe template was subcloned by using primer pair 5’- AAGGATGAGCGCAATGTAGA -3’and 5’- CTGTTCCTGAGTCAGTCCTC -3’. Riboprobes were generated using a 1:3 ratio of UTP and DIG-UTP (Roche). After probe synthesis, we removed unincorporated nucleotides via column filtration according to manufacturer’s protocol (Megaclear, Ambion).

*In situ Hybridization*

Slides were prehybridized with a solution containing 50% formamide, 5X SSC, 5X Denhardt’s solution, 250 µg/ml yeast tRNA, and 500 µg/ml herring sperm DNA for 5 hours at 60°C in a hybridization chamber containing chamber buffer solution (50% formamide, 2X SSC). Then we hybridized the slides overnight at 67°C with fresh prehybridization solution containing 340 ng of *npas4a* antisense or 380 ng of *gabbr1a* riboprobe per slide. Following hybridization we performed two washes in 2X SSC at room temperature for *npas4a* (one wash in 2X SSC at 60^o^C, one wash in 2X SSC at room temperature for *gabbr1a*), then RNase A treated the slides (0.5M NaCl, 10 mM Tris pH 8.0, 2.25 mM EDTA, 0.2 µg/ml RNase A), followed by increasingly stringent washes (2X, 1X, 0.5X, 0.25X SSC) and then a final wash in Buffer B1 (100 mM Tris pH 7.5, 150 mM NaCl). Sections were then incubated overnight at 4°C with Anti-Digoxigenin AP antibody (Roche). After antibody incubation we washed sections twice in Buffer B1 and then blocked endogenous alkaline phosphatase activity with a 30 minute wash in Buffer B3 (100mM Tris pH 9.5, 100 mM NaCl, 50 mM MgCl2, 5 mM levamisole) in the dark. We used colorimetric detection using NBT/BCIP stock solution (Roche). The colorimetric reaction was stopped (80 minutes for *Npas4a* and 12 hours for *Gabbr1a*) by rinsing sections three times in ultrapure type 1 water and then progressively dehydrating sections in ethanol (25%, 50%, 70%, 95%).

*Brain Region Analysis*

The light settings were set to the maximum, and two 1/16 filters were placed over the light source to keep consistency across days. The measuring box was always placed in the middle of the brain region on the dorsal-ventral plane, excluding the midline. We measured the mean intensity bilaterally if available, and averaged all of the intensities for each individual for each brain region. Depending on the size of the brain region, the number of sections averaged per individual ranged from two to six consecutive sections. Consecutive sections were 48 µm apart. The anterior commissure was identified as a landmark for each of the brain regions. We measured the Dm (13003.92 µm^2^) and Dl (13003.92 µm^2^) for 1-2 sections prior to and 3-4 sections following the anterior commissure. We measured the Vv (9907.28 µm^2^) and Vd (9907.28 µm^2^) for 3-4 sections preceding the anterior commissure. We measured the Vs (9907.28 µm^2^) for the slice containing the anterior commissure and 1-2 following it.

**Tables**

**Table S1.** Brain region terminology, abbreviations, and putative tetrapod homologue regions.

| Teleost Region | Abbreviation | Putative Tetrapod Homologue |
| --- | --- | --- |
| Area dorsomedialis telencephali | Dm | Basolateral amygdala |
| Area dorsolateralis telencephali | Dl | Pallial hippocampus |
| Area ventroventralis telencephali | Vv | Lateral septum |
| Area dorsoventralis telencephali | Vd | Striatum |
| Ventralis supracommissuralis telencephali | Vs | Bed nucleus of the stria terminalis |

**Figures**


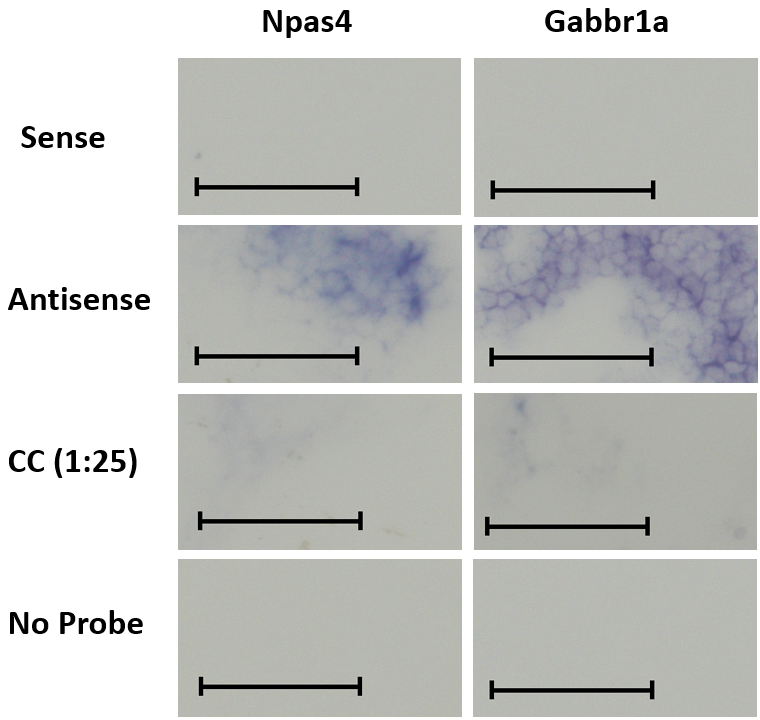


**Figure S1.** Pilot *in situ* hybridization results for the *Npas4a* and *Gabbr1a* genes. There was strong signal in the antisense, proportionally reduced signal in the cold-competitor (1:25 ratio of DIG-labeled to unlabeled riboprobe), and negligible signal in the sense and no probe permutations. Scale bars represent 50 um.

**
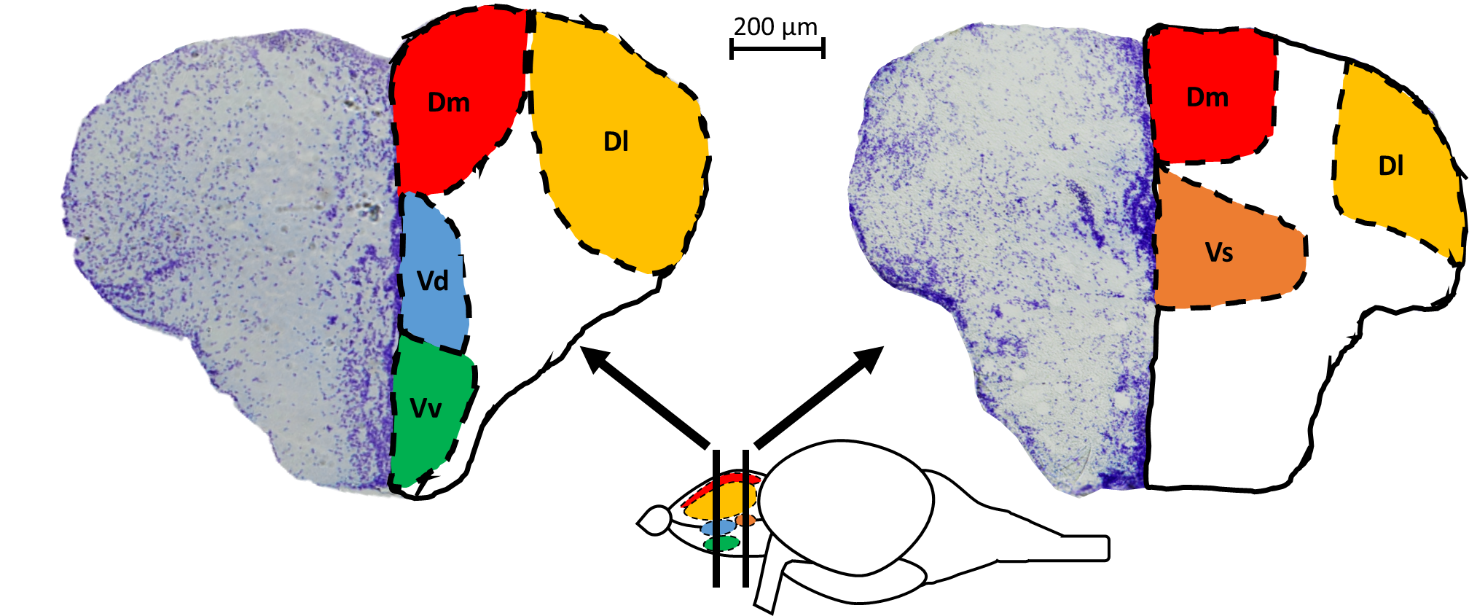
**

**Figure S2.** Standard brain region boundaries used for *in-situ* hybridization analysis of gene expression. Example coronal slices are Nissl stained.


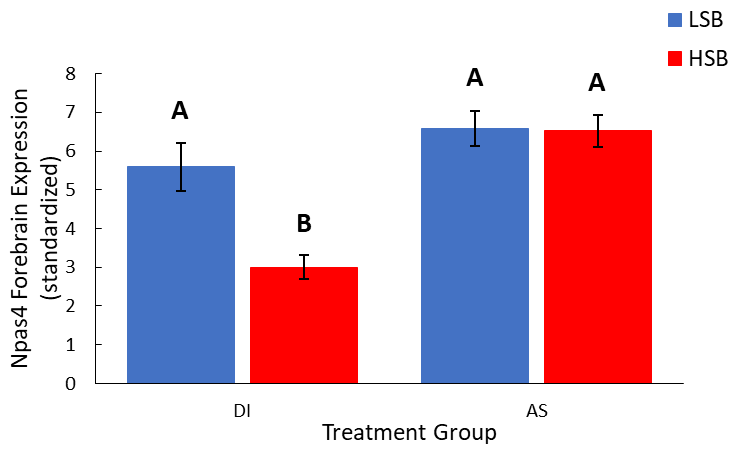

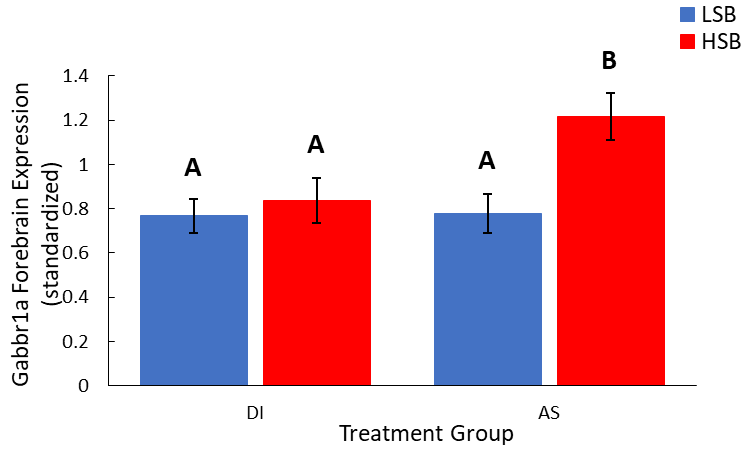


*****

**(B)**

**(A)**

*****

**Figure S3.** *npas4a* (A) and *gabbr1a* (B) forebrain expression standardized to *ef1a*. We measured expression of high stationary behavior (HSB) and low stationary behavior (LSB) fish that were exposed to either alarm substance (AS) or distilled water (DI) during training. Bars represent mean ± 1 SE. Bars labeled with different letters indicate p < .05. * indicates a significant strain main effect.

**
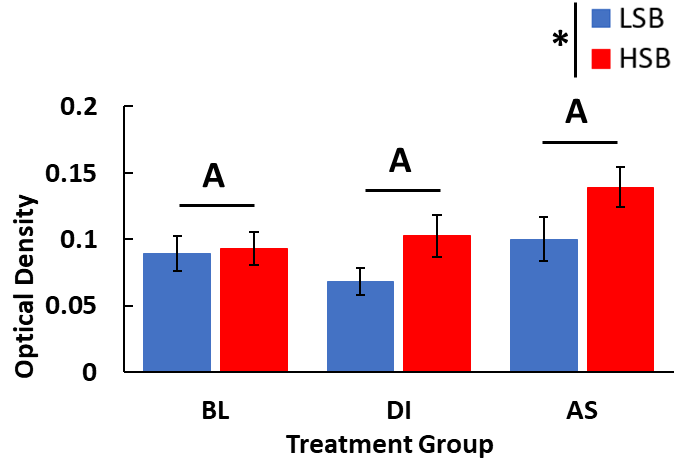
**

**(A)**

**
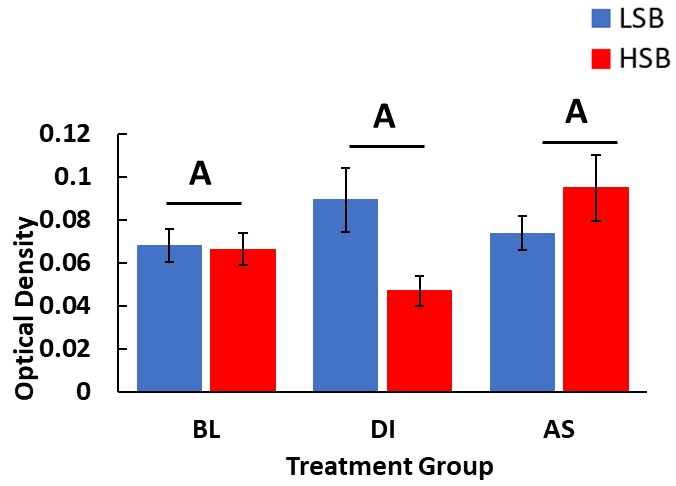
**

**(B)**

**Figure S4.** Expression of *npas4a* in the Vv (A) and Vd (B). We measured expression of high stationary behavior (HSB) and low stationary behavior (LSB) fish at baseline (BL) or exposed to either alarm substance (AS) or distilled water (DI) during training. Bars represent mean ± 1 SE. Bars labeled with different letters indicate p < .05. * indicates a significant strain main effect.


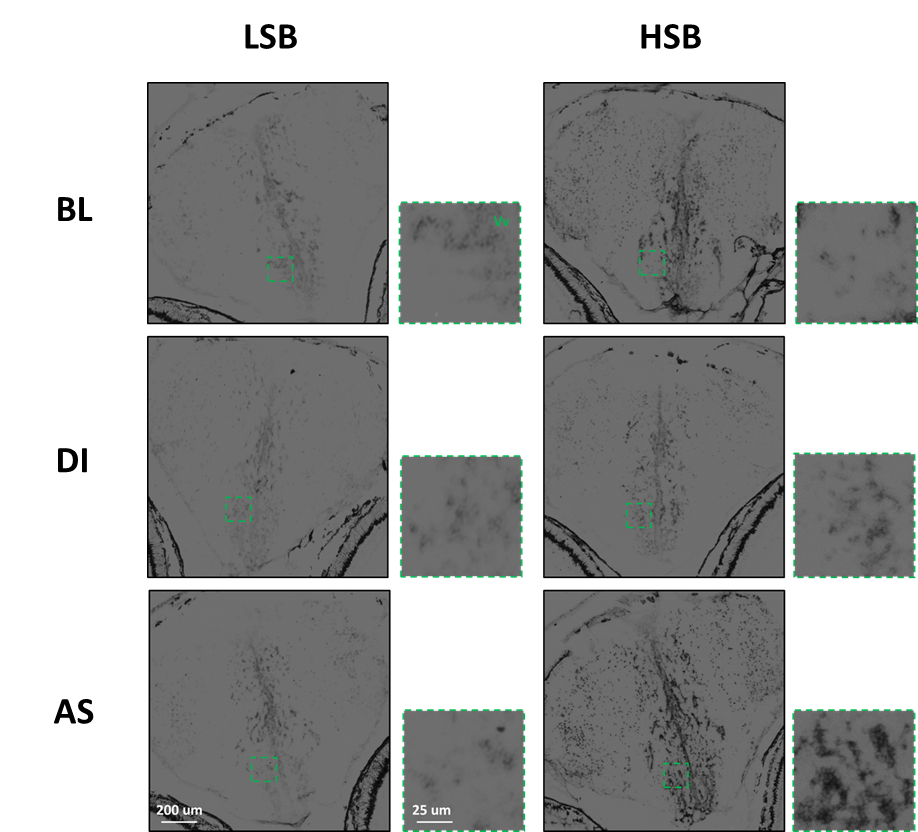


**Figure S5.** Representative images of *Npas4a* expression in the Vv for low stationary behavior (LSB) and high stationary behavior (HSB) fish at baseline (BL) or exposed to either alarm substance (AS) or distilled water (DI) during training.


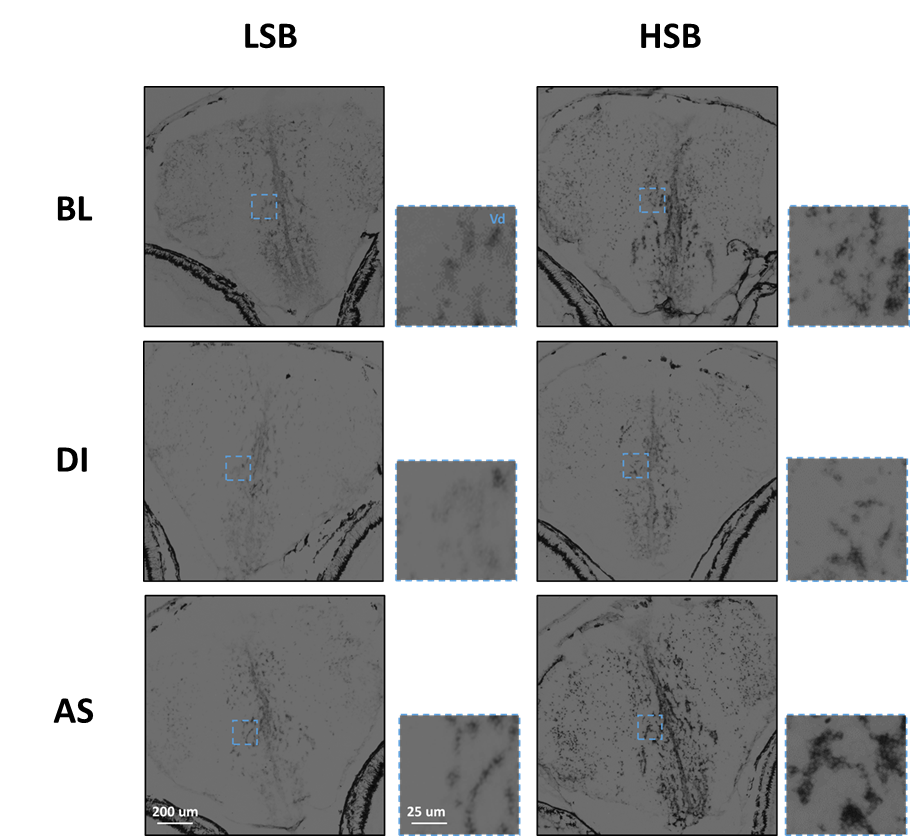


**Figure S6.** Representative images of *Npas4a* expression in the Vd for low stationary behavior (LSB) and high stationary behavior (HSB) fish at baseline (BL) or exposed to either alarm substance (AS) or distilled water (DI) during training.

**
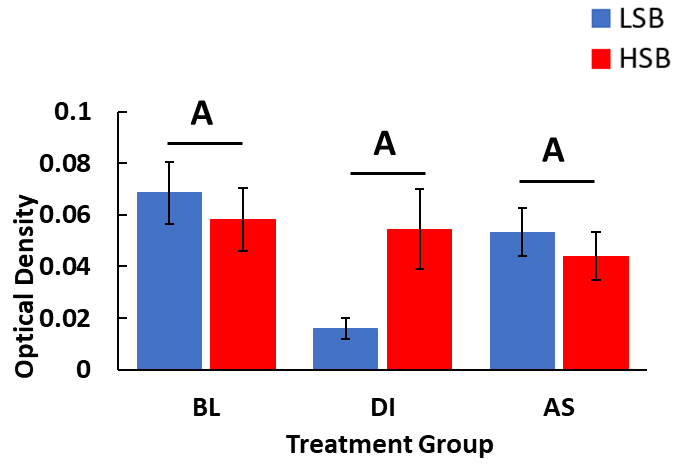

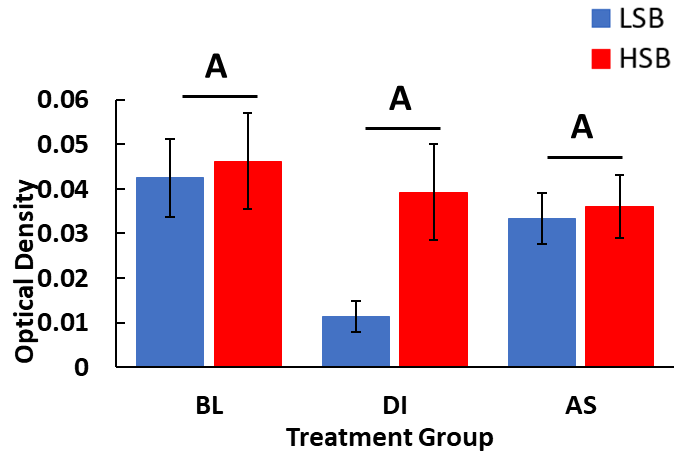
**

**(A)**

**(B)**

**
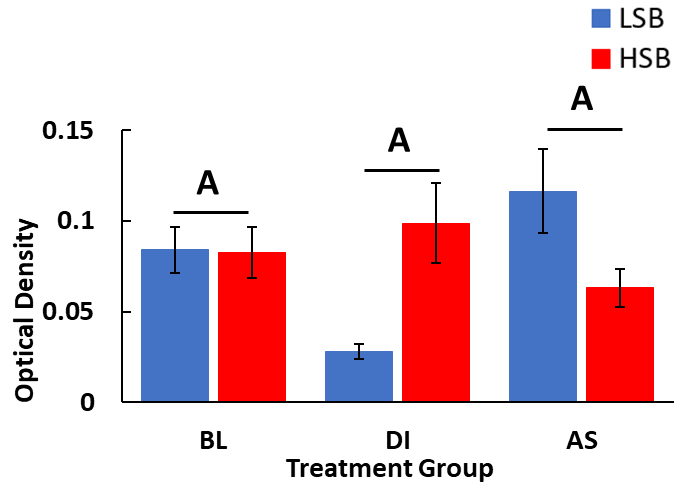

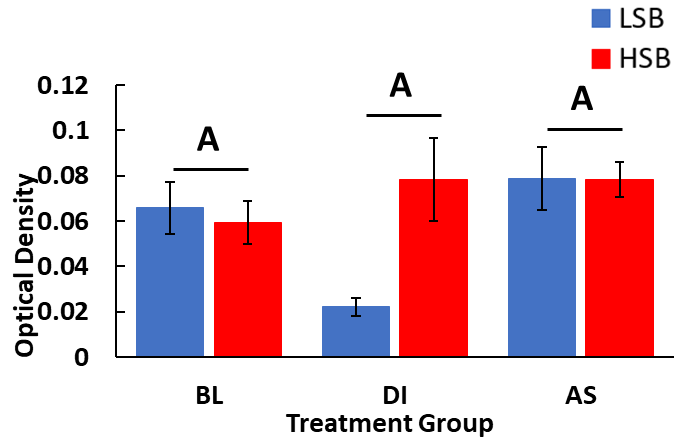
**

**(D)**

**(C)**

**
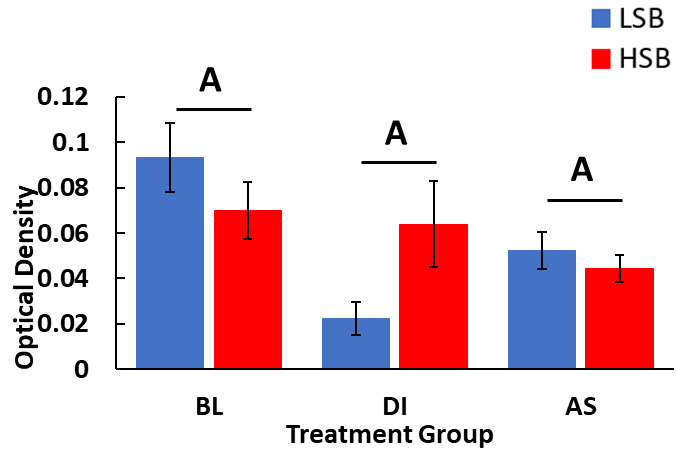
**

**(E)**

**Figure S7.** Expression of *gabbr1a* in the Dm (A), Dl (B), Vv (C), Vd (D), Vs (E). We measured expression of high stationary behavior (HSB; B) and low stationary behavior (LSB; A) fish at baseline (BL) or exposed to either alarm substance (AS) or distilled water (DI) during training. Bars represent mean ± 1 SE. Bars labeled with different letters indicate p < .05. When split by strain, LSB fish exposed to DI water had significantly lower *gabbr1a* OD compared to the baseline and AS groups. There were no treatment group differences in the HSB group.

**
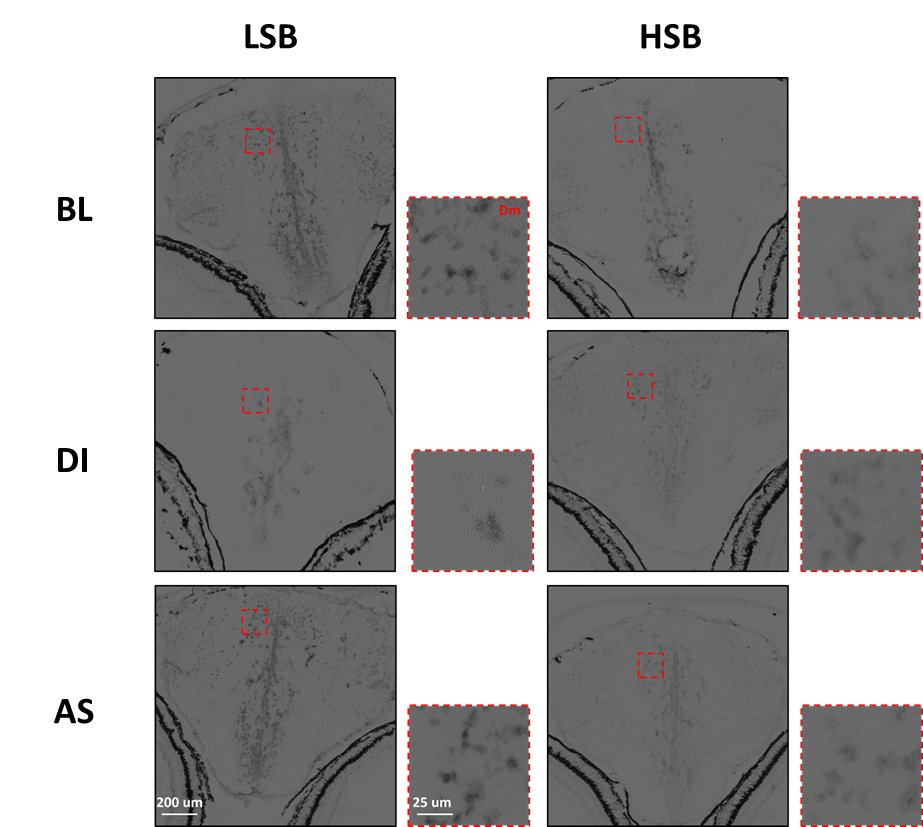
**

**Figure S8.** Representative images of *Gabbr1a* expression in the Dm for low stationary behavior (LSB) and high stationary behavior (HSB) fish at baseline (BL) or exposed to either alarm substance (AS) or distilled water (DI) during training.


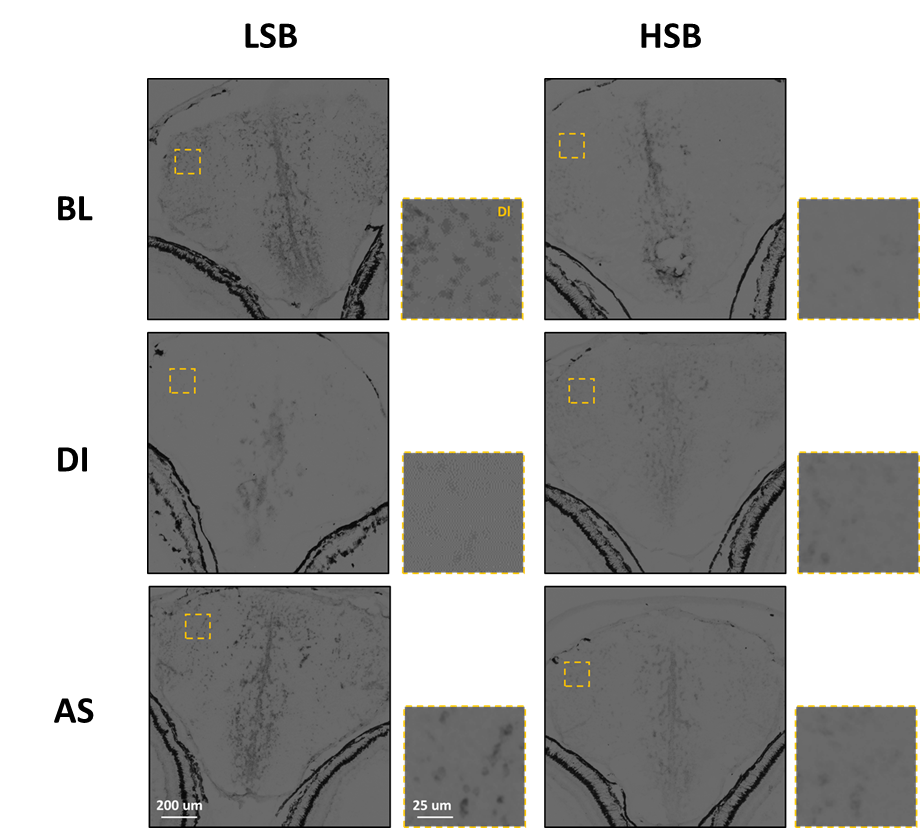


**Figure S9.** Representative images of *Gabbr1a* expression in the Dl for low stationary behavior (LSB) and high stationary behavior (HSB) fish at baseline (BL) or exposed to either alarm substance (AS) or distilled water (DI) during training.


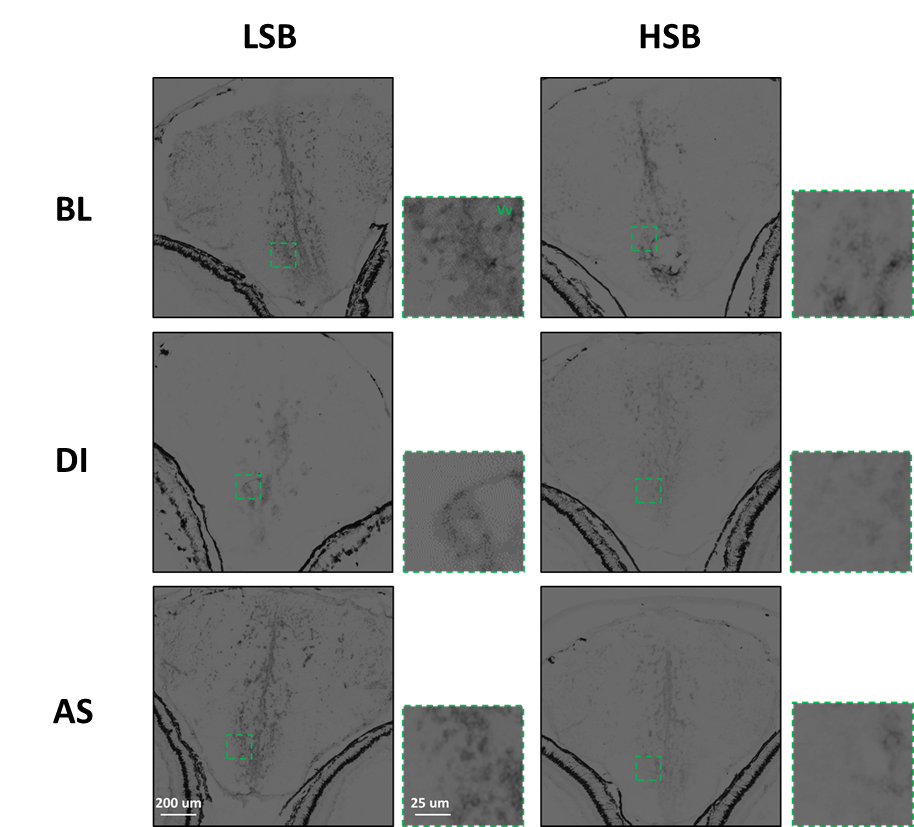


**Figure S10.** Representative images of *Gabbr1a* expression in the Vv for low stationary behavior (LSB) and high stationary behavior (HSB) fish at baseline (BL) or exposed to either alarm substance (AS) or distilled water (DI) during training.


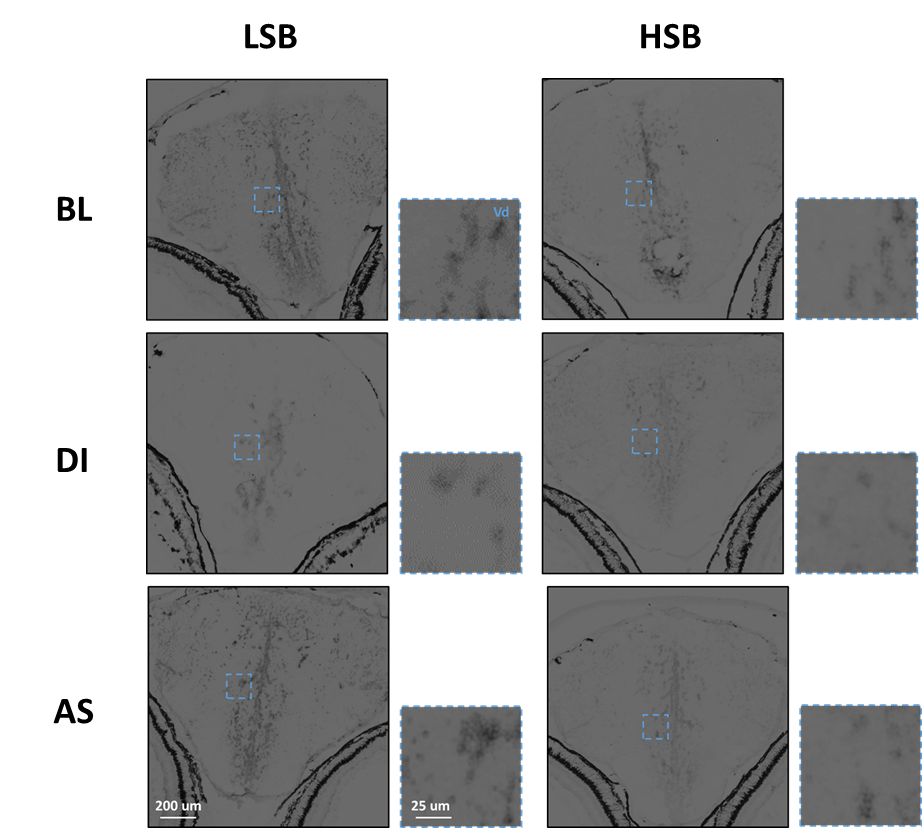


**Figure S11.** Representative images of *Gabbr1a* expression in the Vd for low stationary behavior (LSB) and high stationary behavior (HSB) fish at baseline (BL) or exposed to either alarm substance (AS) or distilled water (DI) during training.


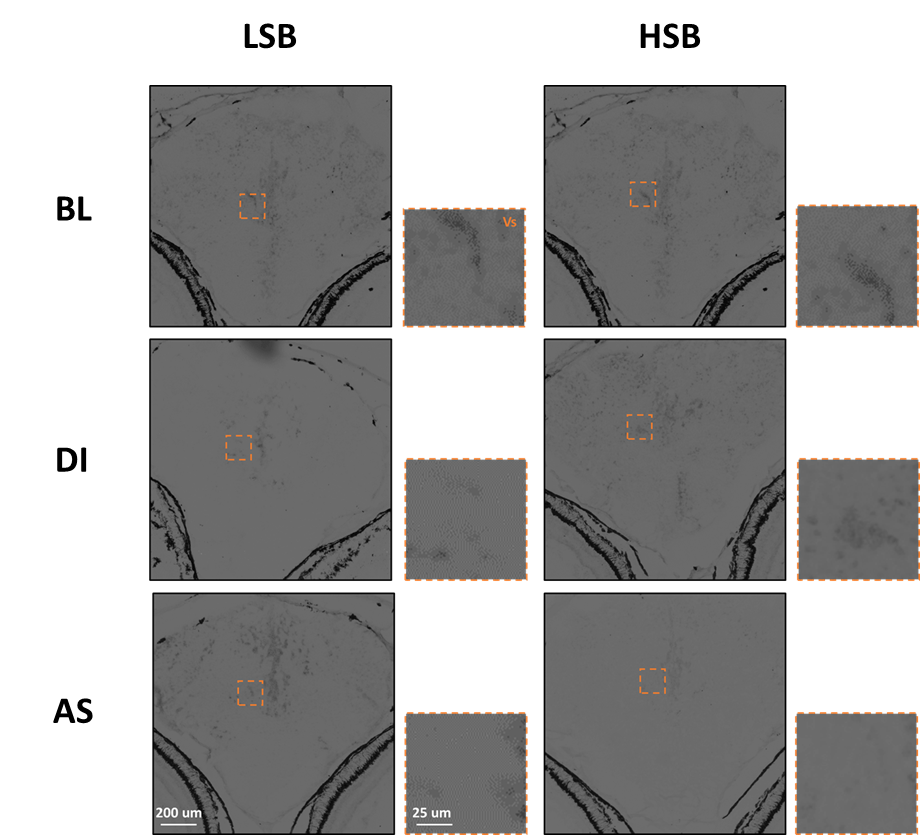


**Figure S12.** Representative images of *Gabbr1a* expression in the Vs for low stationary behavior (LSB) and high stationary behavior (HSB) fish at baseline (BL) or exposed to either alarm substance (AS) or distilled water (DI) during training.
